# Research progress on DNA hydroxymethylation in atrial fibrillation: a review

**DOI:** 10.3389/fphar.2025.1591675

**Published:** 2025-10-08

**Authors:** Shaowei Fan, Jingjing Shi

**Affiliations:** ^1^ Lugouqiao Second Community Health Service Center, China Aerospace Science & Industry Corporation 731 Hospital, Beijing, China; ^2^ Department of Cardiological Medicine, China Academy of Chinese Medical Sciences Guang’anmen Hospital, Beijing, China

**Keywords:** DNA hydroxymethylation, atrial fibrillation, 5-hydroxymethylcytosine, oxidative stress, calcium signaling

## Abstract

Epigenetic modifications play a critical role in the pathogenesis and progression of cardiovascular diseases. Among these, DNA hydroxymethylation has garnered increasing attention in the fields of oncology, hematology, and neurological disorders, serving as a key mechanism for untangling molecular pathways underlying disease etiology. Although emerging evidence has begun to illuminate the role of DNA hydroxymethylation in cardiovascular conditions such as coronary artery disease and atherosclerosis, its implications in atrial fibrillation remain underexplored. This review aims to summarize current understanding and discuss potential mechanisms through which DNA hydroxymethylation may contribute to the development and progression of atrial fibrillation.

## 1 Overview of atrial fibrillation

Atrial fibrillation (AF) is one of the most common clinical arrhythmias, characterized by the loss of regular, organized electrical activity and mechanical contraction in the atria, which are replaced by rapid and disorganized fibrillatory activity. The development of AF is closely associated with multiple factors, including advanced age, sex, obesity, genetic predisposition, and unhealthy lifestyle habits. It is also significantly linked to various comorbidities such as hypertension, coronary artery disease, valvular heart disease, heart failure, diabetes, hyperthyroidism, chronic kidney disease, chronic obstructive pulmonary disease, and metabolic syndrome (Schnabel et al., 2009; Tomaszuk-Kazberuk et al., 2020; Wasmer et al., 2017).

AF poses serious health risks and often leads to severe complications such as heart failure, angina pectoris, myocardial infarction, ischemic stroke, dementia, and even death. It contributes to high rates of disability and mortality, making it a condition of major clinical concern (Benjamin et al., 2019; Alonso et al., 2021).

With the accelerating aging of the global population, the prevalence of AF is increasing annually. Among individuals aged 80 years and older, the prevalence exceeds 10% (Sagris et al., 2021). It is estimated that AF affects approximately 2%–3.4% of the global population (Chugh et al., 2014; Kjerpeseth et al., 2021; Williams et al., 2020; Zoni-Berisso et al., 2014), significantly impairing patients’ quality of life and imposing a substantial economic burden on families and society (Lehto et al., 2022; Colilla et al., 2013; Piccini et al., 2012). For instance, in the United States, the estimated annual medical cost per AF patient ranges between $2,000 and $14,200, with the total national cost for treating AF and its complications exceeding $28 billion per year (Dieleman et al., 2020). According to the Framingham Heart Study, all-cause mortality is 50%–90% higher in patients with AF compared to those without the condition (Alonso et al., 2021).

The pathophysiology of AF is complex and closely related to structural remodeling of the atria, primarily involving loss of cardiac cells and interstitial fibrosis (Iwasaki et al., 2011). Atrial fibrosis serves as a key substrate for the initiation and maintenance of abnormal electrical activity in AF, promoting reentrant arrhythmias (Heijman et al., 2016; Li et al., 1999). The size of the atria and the extent of myocardial fibrosis directly influence treatment outcomes and the risk of complications (Gal and Marrouche, 2017; Zahid et al., 2016). Additionally, oxidative stress, inflammatory responses, abnormalities in mitochondrial energy metabolism, and dysregulation of calcium signaling are recognized as important pathophysiological mechanisms contributing to the onset and progression of AF.

Current clinical management strategies for AF primarily include: restoration and maintenance of sinus rhythm, control of ventricular rate, and prevention of thromboembolic events. The main approaches for restoring sinus rhythm are catheter radiofrequency ablation and antiarrhythmic drug therapy. However, both methods have limitations. Radiofrequency ablation carries a risk of recurrence, while antiarrhythmic drugs may have potential side effects such as pro-arrhythmia. Therefore, further investigation into the pathological mechanisms of AF, along with optimization of diagnostic, therapeutic, and preventive strategies, is essential to provide safer, more effective, and durable solutions for patients.

## 2 Overview of DNA hydroxymethylation

Epigenetics refers to modifications in gene expression that lead to phenotypic changes without alterations in the underlying DNA sequence. Major epigenetic mechanisms include: (1) genomic DNA methylation; (2) modifications of DNA-associated proteins; and (3) regulation by non-coding RNAs (Ameer et al., 2020). Among these, DNA methylation at the 5-position of cytosine has been extensively studied. This process is catalyzed by DNA methyltransferases (DNMTs), which transfer a methyl group (-CH_3_) to the 5′cytosine within CpG islands-regions rich in CpG dinucleotides often located in promoter areas. Methylation at these sites typically inhibits the binding of transcription factors, thereby suppressing gene transcription and influencing phenotypic outcomes.

Closely related to DNA methylation is DNA hydroxymethylation (DNAhm), another key epigenetic modification occurring at the DNA level. DNA hydroxymethylation involves the oxidation of 5-methylcytosine (5mC) to 5-hydroxymethylcytosine (5hmC), a reaction catalyzed by ten-eleven translocation (TET) enzymes in an Fe^2+^- and α-ketoglutarate (α-KG)-dependent manner (Guallar et al., 2018; Tahiliani et al., 2009). 5hmC plays important roles in gene regulation, particularly in cardiac cells during development and under pathological conditions such as hypertrophy (Greco et al., 2016). It is associated with transcriptional activation and can modulate the activity of transcription start sites (TSS) and active enhancers (Ficz et al., 2011; Pastor et al., 2011; Tsagaratou et al., 2014). In the absence of genetic mutations, 5hmC levels are primarily regulated by TET enzyme activity.

Studies by Olsen et al. indicate that ischemic and inflammatory processes-common in conditions such as coronary artery disease-promote oxidative stress, which may lead to oxidative DNA damage and the accumulation of oxidized DNA bases (Olsen et al., 2017; Barzilai and Yamamoto, 2004). 5hmC is implicated in DNA repair mechanisms, particularly the base excision repair (BER) pathway (He et al., 2011; Krokan and Bjørås, 2013). BER involves the removal of damaged bases by DNA glycosylases, creating an apurinic/apyrimidinic (AP) site. This is followed by cleavage by AP endonucleases or phosphodiesterases, with subsequent gap filling by DNA polymerases and ligation by DNA ligases. 5hmC can be further oxidized by TET enzymes to form 5-formylcytosine (5 fC) and 5-carboxylcytosine (5caC), which may be excised by thymine DNA glycosylase (TDG) to regenerate unmodified cytosine. Alternatively, 5hmC can be deaminated by enzymes such as AID/APOBEC, yielding 5-hydroxymethyluracil (5hmU), which is then repaired through glycosylase-mediated pathways involving uracil-DNA glycosylase (UNG) or TDG, ultimately restoring standard bases (Krokan and Bjørås, 2013).

Using selective chemical labeling followed by low-input whole-genome sequencing (hmC-Seal), Dong et al. compared 56 individuals with normal coronary arteries (NCA), 53 with stable coronary artery disease (sCAD), and 58 with acute myocardial infarction (AMI). They found that 5hmC-modified SOX9 regulates fibrosis-related genes activated under ischemic injury, promoting disease progression. Similarly, 5hmC-marked RUNX2 was associated with vascular smooth muscle cell calcification, suggesting both may serve as potential prognostic biomarkers in AMI (Dong et al., 2020).

Notably, 5hmC levels increase absolutely during postnatal maturation of murine cardiomyocytes, consistent with observations in neurons (Münzel et al., 2010; Song et al., 2011; Szulwach et al., 2011; Lister et al., 2013). However, 5hmC content is inversely correlated with proliferative status across tissues (Bachman et al., 2014). Thus, high 5hmC levels in adult cardiomyocytes may reflect their low proliferative capacity and terminally differentiated state.

DNA hydroxymethylation is highly dynamic during cardiac development and disease (Greco et al., 2016). Although 5hmC may attract or repel chromatin remodelers, its functional impact often depends on coexistence with active histone marks-such as H3K79me2, H3K9ac, H3K27ac, or H3K4me3-thereby amplifying their effects. For instance, the fetal gene Myh7, encoding α-myosin heavy chain, undergoes substantial 5hmC loss during cardiac maturation. In hypertrophic cardiomyocytes, enhancers near Myh7 become specifically hydroxymethylated, affecting genes involved in the tricarboxylic acid (TCA) cycle, fatty acid oxidation, and energy production. This promotes myocardial hypertrophy, increasing heart weight, enhancing contractility, and elevating cardiac output-key adaptations supporting heart maturation.

## 3 Potential mechanisms by which DNA hydroxymethylation influences atrial fibrillation

Currently, research on DNA hydroxymethylation in the cardiovascular system remains limited, with very few studies focusing specifically on AF. Existing evidence indicates that TET2 expression is significantly upregulated in peripheral blood mononuclear cells and aortic atherosclerotic plaques of elderly patients with CAD, accompanied by elevated levels of both DNA methylation and hydroxymethylation, which correlate positively with the severity of coronary atherosclerosis (Jiang et al., 2019). A study involving 10 young and 10 elderly healthy women revealed that age-related changes in DNAhm and genes with high DNAhm levels are involved in regulating the immune system during aging (Johnson et al., 2020).

Atrial fibrillation exhibits a strong age-dependent prevalence. Moreover, CAD, which involves varying degrees of myocardial ischemia, contributes to both structural and electrical remodeling of the heart. Consequently, patients with CAD have a higher likelihood of developing AF compared to the general population. During the pathogenesis of AF, mitogen-activated protein kinases (MAPKs)-particularly the extracellular signal-regulated kinase (ERK) 1/2-play crucial roles in cellular proliferation, differentiation, and development. Activation of ERK1/2 occurs through phosphorylation mediated by MAPK/ERK kinase (MEK). Studies have shown that ERK1/2 is activated in cardiac cells exposed to neurohormones such as angiotensin II (Pan et al., 2005; Takahashi et al., 2005; de Jonge et al., 2007). ERK signaling integrates inputs from multiple receptor systems and distal signaling pathways, ultimately promoting cardiac hypertrophy-a finding consistent with clinical observations that hypertension is a strong and independent predictor of AF. According to the Framingham Heart Study, individuals with hypertension have a 1.8-fold higher risk of developing new-onset AF compared to those with normal blood pressure (Benjamin et al., 1994).

**FIGURE 1:**
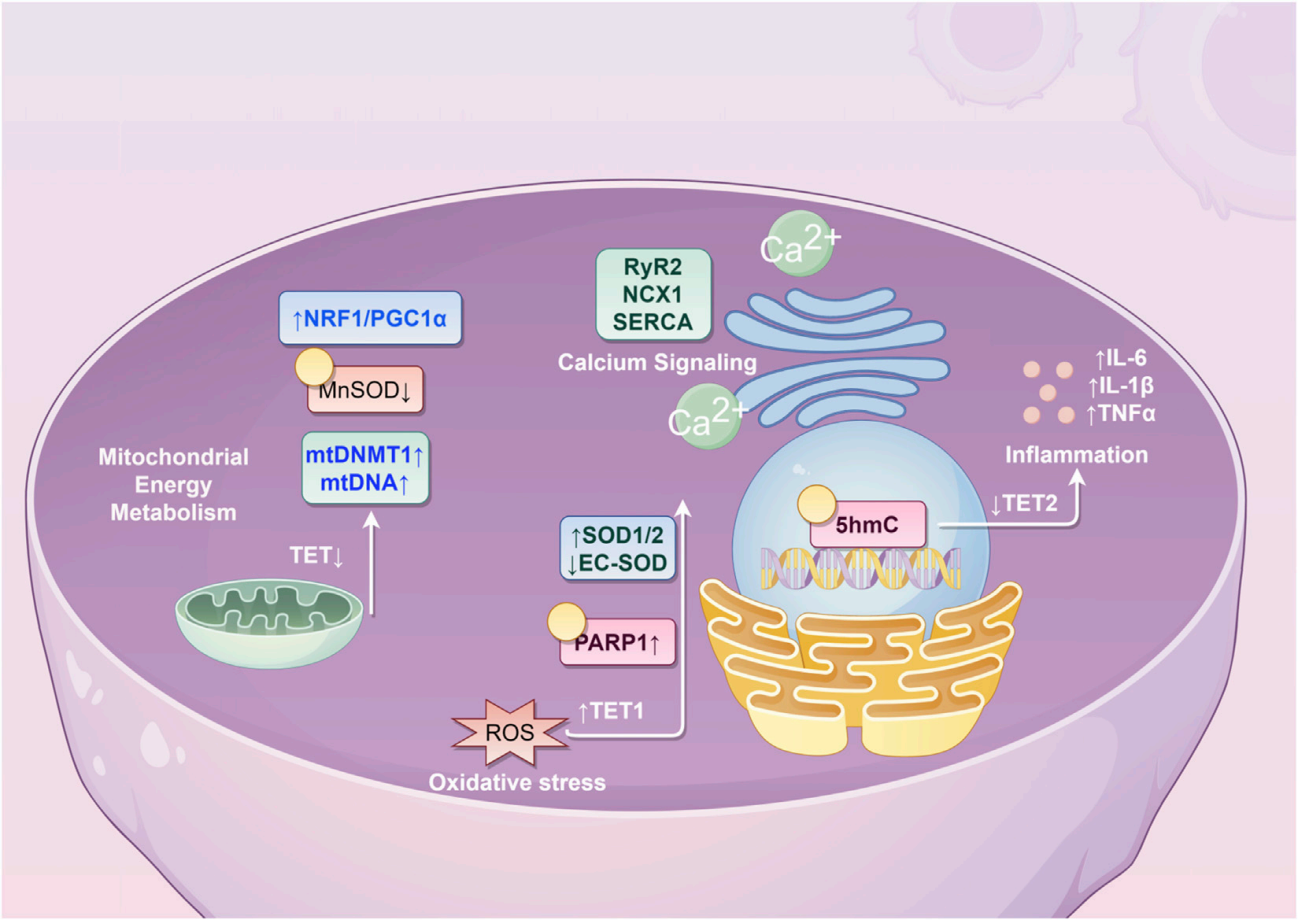
Potential molecular mechanisms underlying the role of 5hmC in atrial fibrillation. (NRF1, nuclear respiratory factor 1; PCG1α, peroxisome proliferator-activated receptor γ coactivator 1α; MnSOD, Manganese Superoxide Dismutase; mtDNMT1, mitochondrial DNA methyltransferase 1; mtDNA, mitochondrial DNA; TET, ten-eleven translocation; RyR2, ryanodine receptor 2; NCX1, sodium-calcium exchanger 1; SERCA, sarco/endoplasmic reticulum calcium ATPase; SOD, superoxide dismutase; EC-SOD, extracellular superoxide dismutase; PARP1, poly(ADP-ribose) polymerase 1; ROS, reactive oxygen species; IL, interleukin; TNF, tumor necrosis factor; 5hmC, 5-hydroxymethylcytosine).

Given these connections, it is plausible that DNA hydroxymethylation may participate in the pathogenesis of AF. This review explores potential mechanisms through the following aspects (Table 1; Figure 1).

**TABLE 1:** Potential molecular mechanisms of 5hmC in atrial fibrillation pathogenesis.

Pathophysiological module	Key epigenetic alterations	Major target genes/Pathways	Functional outcome and role in AF	Primary references
Myocardial hypertrophy and fibrosis	• ↓TET2 → ↓Hspa1b → ↑p-ERK • ↑DNMT/↓TET3 → ↑Methylation of RASAL1 → ↑Ras/ERK signaling • ↑TET2 → ↑5hmC on TGF-β promoter → Fibroblast activation • Altered DNMT/TET expression by maternal environment or cAMP	• ERK/MAPK signaling • TGF-β/Smad signaling • Ras GTPase pathway • Fetal gene program (Nppa, Nppb)	A central mechanism driving structural remodeling. Epigenetic dysregulation activates pro-fibrotic and pro-hypertrophic signaling pathways, leading to excessive extracellular matrix deposition and atrial dilation	Olsen et al. (2017), Tao et al. (2021), Spearman et al. (2018), Niu and Tan (2023), Tabish et al. (2019), Li et al. (2020a), Xu et al. (2015), Xie et al. (2022), Shi et al. (2023), Fang et al. (2015)
Oxidative stress response	• PARP1-mediated ↑TET1 activity and ↑5hmC • TET1-dependent demethylation of SOD1/SOD2 promoters • TNF-α-induced ↓TET1 and ↓5hmC↓ → EC-SOD	• SOD1, SOD2, EC-SOD • ROS signaling pathways	Dual role of TET1/5hmC: Can either increase ROS (via SOD1/2) or reduce antioxidant defense (via EC-SOD). Drives SMC phenotypic switching, endothelial dysfunction, and fibrosis	Zhang et al. (2021), Fan et al. (2023), Morisawa et al. (2017)
Mitochondrial metabolism and function	• TCA cycle metabolites (succinate/fumarate) inhibit TETs → ↓5hmC • Hypoxia-induced ↑mtDNMT1 → ↑mtDNA methylation • MnSOD deficiency → SDH dysfunction → succinate depletion → ↓TET activity → ↓5hmC	• Nuclear genome-wide expression • Mitochondrial DNA-encoded genes • Antioxidant response elements	Establishes a bidirectional crosstalk: Mitochondrial dysfunction alters nuclear epigenetics, which further impairs mitochondrial function. Leads to bioenergetic deficit, excessive ROS production, and cardiomyocyte dysfunction	Xiao et al. (2012), Chouchani et al. (2014), Mills et al. (2016), Brissot et al. (2019), Liu et al. (2020a), Liu et al. (2020b), Pei et al. (2022), Ji et al. (2018), Shock et al. (2011), Korantzopoulos et al. (2007), Melov et al. (1999), Powell and Jackson (2003), Cramer-Morales et al (2020), Liu et al. (2022)
Immune-inflammatory regulation	• ↑5hmC on TNF-α promoter in macrophages • ↓5hmC induced by pro-inflammatory cytokines (IL-1β, TNF-α)	• TNF-α, IL-6, IL-1β • Inflammatory signaling networks	Creates a self-amplifying loop: Epigenetic changes enhance cytokine production, which further disrupts the epigenome. Promotes endothelial damage, thrombogenesis, and electrical instability	Watson et al. (2009), Sethi et al. (2008), Balkwill (2009), Ren et al. (2015), Li et al. (2017), Li et al. (2020b), Sun et al. (2019), Haseeb et al. (2014)
Calcium handling and electrophysiology	• In diabetic cardiomyopathy: ↑DNMT3B, ↑5mC/5hmC on calcium signaling pathways • Potential epigenetic regulation of key Ca^2+^ handling genes is hypothesized but not yet proven in AF.	• RyR2, NCX1, SERCA2a, Cav1.2 (inferred) • Rap1, apelin, PI3K signaling	Proposed link between epigenetic changes and sarcoplasmic reticulum Ca^2+^ leak, promoting delayed afterdepolarizations (DADs) and triggered activity that initiate AF. This represents a critical knowledge gap.	Hove-Madsen et al. (2004), Wang et al. (2021), Yao et al. (2018), Heijman et al. (2020), Dhat et al. (2023)

Abbreviations: 5hmC, 5-hydroxymethylcytosine; 5mC, 5-methylcytosine; AF, atrial fibrillation; cAMP, cyclic adenosine monophosphate; Cav1.2, L-type calcium channel; DNMT, DNA methyltransferase; EC-SOD, extracellular superoxide dismutase; ERK, extracellular signal-regulated kinase; IL, interleukin; MAPK, mitogen-activated protein kinase; MnSOD, Manganese Superoxide Dismutase; mtDNA, mitochondrial DNA; mtDNMT1, mitochondrial DNA methyltransferase 1; NCX1, sodium-calcium exchanger 1; PARP1, poly (ADP-ribose) polymerase 1; PI3K, phosphoinositide 3-kinase; ROS, reactive oxygen species; RyR2, ryanodine receptor 2; SDH, succinate dehydrogenase; SERCA2a, sarco/endoplasmic reticulum calcium ATPase; SMC, smooth muscle cell; SOD, superoxide dismutase; TCA, tricarboxylic acid; TET, ten-eleven translocation; TGF-β, transforming growth factor beta; TNF-α, tumor necrosis factor alpha.

### 3.1 5hmC in cardiac hypertrophy and fibrosis

Cardiac hypertrophy and fibrosis represent key pathophysiological mechanisms underlying the development and progression of atrial fibrillation. Cardiac hypertrophy refers to the enlargement of cardiomyocytes in response to sustained physiological or pathological stimuli, serving to maintain cardiac reserve and output. Myocardial fibrosis, a central feature of chronic ischemic heart disease, often results from atherosclerotic coronary artery stenosis leading to moderate-to-severe ischemia. This condition promotes both cardiomyocyte hypertrophy and collagen fiber proliferation, perpetuating a cycle of ischemic injury and hypoxia that may ultimately progress to heart failure, increased mortality, and reduced quality of life.

Studies indicate that TET enzymes and 5hmC play dynamic roles in postnatal cardiac development, with TET2 being the predominant dioxygenase in the heart (Tao et al., 2021). Knock out of TET2 leads to reduced hydroxymethylation in the cardiac genome, altered transcriptomic profiles, and manifestations of cardiac dysfunction, progressive hypertrophy, and fibrosis. This may be attributed to diminished expression of Hspa1b-a regulator of the ERK pathway-resulting in enhanced ERK phosphorylation, pathway hyperactivation, and subsequent induction of cardiomyocyte hypertrophy. Spearman et al. reported that abnormal maternal conditions lead to decreased expression of TET1-3 and DNMT3a, promoting myocardial fibrosis in adult offspring (Spearman et al., 2018). Furthermore, Niu et al. demonstrated that TET2 upregulates 5hmC modification in the TGF-β promoter region, enhancing fibroblast proliferation (Niu and Tan, 2023).

A genome-wide profiling study of DNA hydroxymethylation in a murine model of dilated cardiomyopathy identified over 2000 genes with differential 5hmC modifications, which were enriched in pathways related to inflammation, tissue fibrosis, cell death, cardiac remodeling, cardiomyocyte growth and differentiation, and sarcomere organization (Tabish et al., 2019). Given the clinical coexistence of dilated cardiomyopathy and AF, these pathways may contribute to the pathophysiology of AF, suggesting a potential mechanistic link mediated by 5hmC.

Li et al. reported that hypermethylation of Ras protein activator-like 1 (RASAL1) and Ras association domain family 1 (RASSF1) leads to their downregulation, subsequent activation of the Ras/ERK pathway, and promotion of cardiac fibrosis (Li et al., 2020a). Similarly, Xu et al. found that TGFβ1 upregulates RASAL1 promoter methylation, suppresses its expression, increases Ras-GTP activity and endothelial–mesenchymal transition (EndMT), thereby exacerbating fibrosis (Xu et al., 2015). Notably, TET3, an enzyme promoting hydroxymethylation, was significantly reduced in fibrotic cardiomyocytes. These findings suggest that a balance between promoter methylation and hydroxymethylation of RASAL1 plays a critical role in regulating cardiac fibrosis and remodeling.

Moreover, NEIL3 expression is elevated in cardiomyocytes of heart failure patients and post-myocardial infarction mice, particularly in fibroblast-rich regions involved in proliferation, differentiation, extracellular matrix regulation, and scar formation (Olsen et al., 2017). NEIL3-dependent DNA methylation and hydroxymethylation collectively modulate cardiac fibroblast proliferation and contribute to structural remodeling. Chronic catecholamine-induced activation of the cAMP pathway also promotes cardiac hypertrophy and fibrosis. Xie et al. showed that reduced hydroxymethylation in the miR-3571 promoter downregulates its expression, upregulates claudin 1 (CLDN1) and ERK1/2, and facilitates vascular smooth muscle cell proliferation and migration, thereby contributing to cardiovascular pathogenesis (Xie et al., 2022).

Under hypoxic conditions, reduced TET2 expression in vascular endothelial cells impairs its DNA demethylase activity at specific STAT3 target gene promoters, inhibiting the STAT3 pathway and angiogenesis, which consequently impedes blood flow recovery, reduces capillary density, and promotes cardiac fibrosis and remodeling (Shi et al., 2023). Fang et al. demonstrated that the stable cAMP analog DBcAMP alters the expression of DNMTs and TETs, increases DNA methylation in cardiomyocytes, and upregulates markers of hypertrophy such as Myh6, Myh7, Myh7b, Tnni3, ANP, BNP, Gata4, Mef2c, Mef2d, Nfatc1, miR208a, and miR208b (Fang et al., 2015). However, changes in 5hmC levels were not assessed in this study.

Given the interplay between DNA methylation and hydroxymethylation in regulating myocardial fibrosis, further investigation using atrial fibrillation animal models may help elucidate the role of 5hmC in the pathophysiology of atrial fibrillation. Such studies could enhance our understanding of the disease and inform novel therapeutic and preventive strategies.

### 3.2 5hmC in oxidative stress

During the development of atherosclerosis, vascular smooth muscle cells (SMCs) undergo a phenotypic transition from a contractile to a synthetic state. Studies have shown that oxidative stress activates poly (ADP-ribose) polymerase 1 (PARP1), which in turn promotes the expression of TET1 and increases PARylation-dependent 5hmC levels, contributing to vascular remodeling (Zhang et al., 2021). Furthermore, Fan et al. demonstrated that TET1 downregulates 5mC levels in the promoters of superoxide dismutase (SOD) 1 and SOD2, thereby enhancing their expression. This leads to the accumulation of reactive oxygen species (ROS), induction of G2/M cell cycle arrest, and promotion of inflammation and fibrosis (Fan et al., 2023).

Extracellular superoxide dismutase (EC-SOD) is a secreted antioxidant enzyme predominantly localized in the vascular wall, where it plays a protective role against oxidative stress by safeguarding vascular endothelial function. Evidence suggests that tumor necrosis factor-alpha (TNF-α) markedly suppresses TET1 expression in fibroblasts, thereby altering DNA hydroxymethylation and significantly reducing EC-SOD levels. This process exacerbates vascular endothelial injury and accelerates the progression of cardiovascular diseases (Morisawa et al., 2017).

### 3.3 5hmC in mitochondrial energy metabolism

TCA cycle is central to mitochondrial redox reactions that generate energy and support cardiac cell function. Key intermediates of this cycle, fumarate and succinate, act as competitive inhibitors of α-KG-dependent enzymes that regulate DNA hydroxymethylation levels (Xiao et al., 2012). Fe(II), α-KG, fumarate, and succinate play crucial roles in maintaining mitochondrial function (Chouchani et al., 2014; Mills et al., 2016; Brissot et al., 2019). Elevated levels of fumarate and succinate in the TCA cycle inhibit TET enzyme activity, leading to dysregulation of DNA hydroxymethylation (Liu et al., 2020a; Liu et al., 2020b). On one hand, the mitochondrial TCA cycle modulates 5hmC levels by influencing the activity of TET enzymes, thereby affecting cardiac function (Pei et al., 2022). On the other hand, fumarate, succinate, and α-KG are vital intermediates for mitochondrial integrity; hence, mitochondrial dysfunction suppresses TET activity and disrupts DNA hydroxymethylation (Ji et al., 2018).

Similar to the nuclear genome, mitochondrial DNA (mtDNA) also undergoes DNA methylation and hydroxymethylation. Mitochondrial DNA methyltransferase 1 (mtDNMT1), present in the mitochondrial matrix, binds to mtDNA and facilitates its methylation. Studies indicate that under hypoxic conditions, transcription factors nuclear respiratory factor 1 (NRF1) and peroxisome proliferator-activated receptor γ coactivator (PGC) 1α upregulate mtDNMT1, enhancing mtDNA methylation and modulating mitochondrial function (Shock et al., 2011). Manganese superoxide dismutase (MnSOD) catalyzes the dismutation of superoxide (O_2_•^−^) into hydrogen peroxide (H_2_O_2_), protecting mitochondria from oxidative damage. Its expression correlates positively with oxidative stress levels (Korantzopoulos et al., 2007). As atrial fibrosis progresses, MnSOD secretion by atrial cardiac cells decreases. Cells deficient in MnSOD exhibit elevated O_2_•^−^ levels, loss of redox homeostasis, inactivation of iron-sulfur (Fe-S) clusters, and reduced succinate dehydrogenase (SDH) activity (Melov et al., 1999; Powell and Jackson, 2003).

Succinate serves as a direct regulator of TET enzyme activity. Cramer-Morales et al. demonstrated that deficiency in MnSOD leads to downregulation of SDH activity, resulting in reduced succinate levels and impaired TET function. This decrease in genomic DNA hydroxymethylation is accompanied by a corresponding increase in DNA methylation, ultimately contributing to abnormalities in cardiac electrical conduction and contractility (Cramer-Morales et al., 2020). A case-control study by Liu et al. further reported that MnSOD levels are significantly elevated in patients with paroxysmal AF compared to both non-AF controls and those with persistent AF, suggesting that MnSOD may serve as an independent risk factor for paroxysmal AF (Liu et al., 2022).

However, research on mtDNA hydroxymethylation in the context of AF progression remains limited. In cardiovascular diseases such as AF and heart failure, mitochondrial function in cardiac cells is a focal point of investigation. Alterations in myocardial energy supply directly affect contractility, cardiac output, and consequently, both systemic and coronary perfusion. Therefore, exploring dynamic changes in mtDNA hydroxymethylation may offer novel insights into myocardial mitochondrial energy metabolism, quality control mechanisms, and reveal new pathophysiological pathways involved in AF, potentially informing future therapeutic and preventive strategies.

### 3.4 5hmC in the regulation of immune-inflammatory responses

Multiple inflammatory markers and mediators-including C-reactive protein (CRP), TNF-α, interleukin (IL)-2, IL-6, IL-8, and monocyte chemoattractant protein (MCP)-1-are implicated in the pathogenesis of AF. These molecules may contribute to AF progression by promoting endothelial injury, activating prothrombin, and enhancing platelet activation (Watson et al., 2009).

TNF-α, a glycoprotein hormone consisting of 185 amino acids, is primarily synthesized by monocytes and macrophages. As a key mediator under pathophysiological conditions, TNF-α can either induce or suppress the production of various inflammatory molecules-such as cyclooxygenases, matrix metalloproteinases, and cytokines-thereby driving inflammatory progression (Sethi et al., 2008; Balkwill, 2009). Previous studies have demonstrated that TNF-α is involved in the pathophysiology of AF (Ren et al., 2015; Li et al., 2017; Li et al., 2020b). Elevated levels of TNF-α are observed in AF patients and correlate positively with left atrial diameter and AF duration. Moreover, TNF-α serves as a significant predictor of adverse outcomes, including ischemic stroke.

Notably, during the differentiation of monocytes into macrophages and following lipopolysaccharide (LPS) stimulation, the 5hmC level at the TNF-α promoter region increases specifically, leading to the upregulation of TNF-α expression (Sun et al., 2019). Furthermore, studies by Haseeb et al. indicate that proinflammatory cytokines such as IL-1β and TNF-α can downregulate DNA hydroxymethylation levels, thereby modulating gene expression and contributing to immune-inflammatory regulation, which in turn promotes the development of AF (Haseeb et al., 2014).

### 3.5 5hmC in calcium signaling

Spontaneous calcium leakage from the sarcoplasmic reticulum in cardiac cells may underlie triggered electrical activity and contribute to the pathogenesis of AF (Hove-Madsen et al., 2004). Abnormal calcium release, often manifested as increased frequency of calcium sparks and calcium waves (SCaWs), promotes spontaneous efflux of calcium through ryanodine receptor 2 (RyR2) channels, subsequently activating the sodium–calcium exchanger (NCX1). The electrogenic exchange of one calcium ion for three sodium ions via NCX1 generates a transient inward depolarizing current, which may serve as a trigger for AF (Wang et al., 2021). Yao et al. demonstrated that AF is associated with enhanced activation of the NLRP3 inflammasome in atrial cardiomyocytes, which can lead to ectopic electrical activity, aberrant sarcoplasmic reticulum calcium release, shortened atrial effective refractory period, and atrial hypertrophy (Yao et al., 2018; Heijman et al., 2020).

In diabetic cardiomyocytes, upregulation of DNMT3B, MBD2, and MeCP2 has been observed, accompanied by accumulation of both 5mC and 5hmC (Dhat et al., 2023). Calcium signaling was identified as one of the pathways most significantly affected by these DNA methylation and hydroxymethylation modifications. Genomic regions with hypermethylation were enriched in genes related to Rap1, apelin, and phosphatidylinositol signaling, whereas metabolic pathways were most strongly influenced by hyperhydroxymethylation. Nevertheless, the roles of DNA hydroxymethylation and methylation in calcium signaling specifically in the context of AF remain underexplored and warrant further investigation.

## 4 Conclusion

Current research on 5hmC in the context of AF remains limited. However, significant progress has been made in understanding its role in related cardiovascular and cerebrovascular diseases, including heart failure, atherosclerosis, coronary artery disease, cerebrovascular disease, and hypertension. These advances may provide valuable insights into the epigenetic mechanisms underlying AF.

The recent development of innovative sequencing technologies for 5hmC-such as the selective chemical labeling method (hmC-Seal)-offers powerful tools for elucidating the epigenetic features of AF. For instance, the application of hmC-Seal to circulating cell-free DNA (cfDNA) enables precise mapping of hydroxymethylated sites, facilitating disease diagnosis and prediction. This approach holds promise for identifying diagnostic biomarkers, thereby reducing the need for invasive procedures and improving patient convenience.

Furthermore, a deeper understanding of 5hmC dynamics may contribute to the development of targeted epigenetic therapies aimed specifically at modulating DNA hydroxymethylation. Such strategies could potentially inhibit the initiation and progression of AF, ultimately reducing its incidence, disability, and mortality rates.
